# Responsiveness of the Post-Concussion Symptom Scale to Monitor Clinical Recovery After Concussion or Mild Traumatic Brain Injury

**DOI:** 10.1177/23259671221127049

**Published:** 2022-10-12

**Authors:** Pierre Langevin, Pierre Frémont, Philippe Fait, Jean-Sébastien Roy

**Affiliations:** * Clinique Cortex and Physio Interactive, Quebec City, Québec, Canada.; † Department of Rehabilitation, Faculty of Medicine, Université Laval, Quebec City, Québec, Canada.; ‡ Centre for Interdisciplinary Research in Rehabilitation and Social Integration (CIRRIS), Québec Rehabilitation Institute, Quebec City, Québec, Canada.; § Department of Human Kinetics, Université du Québec à Trois-Rivières, Quebec City, Québec, Canada.; ∥ Research Center in Neuropsychology and Cognition (CERNEC), Montréal, Québec, Canada.

**Keywords:** concussion, persistent postconcussive symptoms, responsiveness, Post-Concussion Symptoms Scale

## Abstract

**Background:**

The Post-Concussion Symptom Scale (PCSS) is used to assess the number and intensity of symptoms after a concussion/mild traumatic brain injury. However, its responsiveness to monitor clinical recovery has yet to be determined.

**Purpose:**

To evaluate the responsiveness of the PCSS to change and longitudinal validity in patients with persistent postconcussive symptoms as well as to explore the responsiveness of other clinical outcome measures to monitor recovery of physical symptoms in patients with persistent postconcussive symptoms.

**Study Design:**

Cohort study (diagnosis); Level of evidence, 2.

**Methods:**

Patients with persistent symptoms after a concussion (N = 109) were evaluated using self-reported questionnaires at baseline and after a 6-week rehabilitation program. The program consisted of an individualized symptom-limited aerobic exercise program combined with education. Questionnaires included the PCSS, Neck Disability Index (NDI), Headache Disability Inventory (HDI), Dizziness Handicap Inventory (DHI), and Numeric Pain Rating Scale (NPRS) related to 1) neck pain and 2) headache. Internal responsiveness was evaluated using the effect size (ES) and standardized response mean (SRM), and external responsiveness was determined with the minimal clinically important difference (MCID) calculated using a receiver operating characteristic curve. The global rating of change was used as the external criterion. Pearson correlations were used to determine the longitudinal validity.

**Results:**

The PCSS was highly responsive (ES and SRM, >1.3) and had an MCID of 26.5 points (of 132) for the total score and 5.5 (of 22) for the number of symptoms. For longitudinal validity, low to moderate correlations were found between changes in PCSS and changes in NDI, HDI, and DHI. The NDI, HDI, DHI, and NPRS were also highly responsive (ES and SRM, >0.8).

**Conclusion:**

All questionnaires including the PCSS were highly responsive and can be used with confidence by clinicians and researchers to evaluate change over time in a concussion population with persistent symptoms.

Concussion or mild traumatic brain injury occurs frequently in contact sports but also in other types of traumatic events, such as falls, car accidents, and physical violence.^5,13,27^ A concussion is defined as a complex pathophysiological process affecting the brain produced by external forces transmitted to the head. It results in a wide assortment of symptoms (eg, physical, cognitive, somatic, emotional),^24,26^ with headache, dizziness, and neck pain being frequently reported.^4,18,41^ While concussion-related symptoms gradually improve within a week in most cases,^27^ persistent postconcussive symptoms can be observed in 21% to 46% of adults 3 to 6 months after the injury.^6,10,40^

Symptoms and disability should be objectively documented to monitor a patient’s status and progress over time in order to guide clinical decision-making.^36^ The Post-Concussion Symptom Scale (PCSS), also known as the 22-Item Post-Concussion Scale,^19^ is a self-reported questionnaire that was recommended at the 5th International Conference on Concussion in Sport^27^ to monitor clinical recovery. It is widely used by health care professionals to document the number and intensity of symptoms after a concussion.^23,9^ Normative data, test-retest reliability (intraclass correlation coefficient [ICC], 0.62-0.69),^23,29^ internal consistency (*r* = 0.93), and minimal detectable change (MDC; total score of 12.3 points) of the PCSS have already been established.^23^ Responsiveness to change, however, has yet to be determined.

Other outcome measures used to measure recovery of physical symptoms after a concussion include the Numeric Pain Rating Scale (NPRS) to measure the intensity of symptoms, especially headache and neck pain,^30^ as well as the Neck Disability Index (NDI),^25^ Headache Disability Inventory (HDI),^14^ and Dizziness Handicap Inventory (DHI),^39^ as they assess commonly described symptoms and disabilities. The responsiveness of these questionnaires has also never been established in people with persistent postconcussive symptoms.

Responsiveness is the capacity of a measure to accurately detect meaningful changes in a patient’s condition.^34^ There are 2 forms of responsiveness: internal and external.^3,12^ Internal responsiveness does not require an external marker of change and can be determined by effect size (ES) and standardized response mean (SRM).^3,12^ External responsiveness requires an external marker of meaningful change and is calculated by different statistical methods such as receiver operating characteristic (ROC) curve to derive a minimal clinically important difference (MCID).^12^

The primary objective of this study was to determine the internal and external responsiveness of the PCSS to monitor clinical recovery after a rehabilitation program in patients with persistent postconcussive symptoms. Secondary objectives were to evaluate the longitudinal validity of the PCSS compared with other questionnaires and to explore the internal responsiveness of the NPRS, NDI, DHI, and HDI for a population with persistent symptoms after a concussion. Since time and rehabilitation interventions have been shown to lead to large improvements in this population,^10,21,37,40^ the a priori hypotheses were that (1) the PCSS would be highly responsive (ES and SRM, >0.8); (2) the area under the ROC curve (AUC) of the PCSS MCID would be ≥0.7^35^; (3) change scores on the PCSS and change scores on the NDI, DHI, and HDI would be positively and moderately correlated (>0.5), while they would not be correlated with change scores on NPRS because NPRS evaluates a single construct and symptom; and (4) HDI, NDI, DHI, and NPRS would be highly responsive (>0.8).

## Methods

### Study Design and Population

This was a prospective cohort study evaluating participants with persistent postconcussive symptoms before and after a 6-week physical rehabilitation program. The study protocol received research ethics committee approval. Adults aged between 18 and 65 years with a concussion diagnosis based on the definition of McCrory et al^27^ were recruited through Quebec City multidisciplinary concussion clinics, medical clinics, and e-blasts at the local university (Université Laval) between September 2019 and June 2021. The study inclusion criteria were (1) concussion within the past 12 weeks with ongoing symptoms including at least dizziness, neck pain, and/or headaches started <72 hours after the trauma; and (2) at least 1 cognitive symptom that started <72 hours after the trauma. Potential participants were excluded if they had (1) >30 minutes of loss of consciousness, (2) ≥24 hours of posttraumatic amnesia, (3) a Glasgow Coma Scale score <13 more than 30 minutes after injury, 4) radiologic evidence of severe brain injury such as subdural hemorrhage, 5) postinjury hospitalization >48 hours, 6) fracture, 7) another associated neurological condition, and 8) cardiovascular or respiratory comorbidities. Based on the COnsensus-based Standards for the selection of health Measurement INstruments (COSMIN) guideline,^31^ a minimum of 100 participants were required for responsiveness analysis.

### Intervention

All participants took part in a 6-week individualized symptom-limited aerobic exercise program supervised by a physical therapist or kinesiologist. The program also included education sessions provided by a neuropsychologist. The program is described thoroughly in a previous publication.^20^

### Evaluations

Participant evaluations were performed on an online secured platform (LimeSurvey; https://www.limesurvey.org) and were managed by an evaluator not involved in the rehabilitation program. At baseline, a link was sent to participants to complete a sociodemographic questionnaire and the study questionnaires (PCSS, NPRS neck pain, NPRS headache, NDI, HDI, HDI). Immediately after the 6-week intervention period, another link was sent to participants with the same study questionnaires and a global rating of change (GRC) question, in which participants rated the overall change in their condition since the initial evaluation on a 15-point scale (range, –7 [a very great deal worse] to +7 [a very great deal better]).^16^

### Outcome Measures: Self-Reported Questionnaires

#### Post-Concussion Symptom Scale

The severity and impact of symptoms was measured by the PCSS.^23^ The PCSS consists of a list of 22 symptoms for which participants rate the intensity from 0 (none) to 6 (severe). A total score was then calculated, with a maximum of 132 points. We also recorded the number of symptoms (of 22) that were rated as an intensity ≥1.

#### Neck Disability Index

The NDI evaluates the neck pain–related disability. The reliability (ICC, 0.73-0.98), construct validity, and responsiveness to change have been demonstrated in various populations but not in a population after a concussion.^25^ The score in percentage was recorded.

#### Headache Disability Inventory

The HDI evaluates the headache-related level of disability. The test-retest reliability (*r* = 0.79-0.83) and the MDC (16 points) are known for populations with migraines.^15^ The score (of 100 points) was recorded.

#### Dizziness Handicap Inventory

The DHI evaluates disability linked to dizziness-like symptoms.^14^ The questionnaire has demonstrated high test-retest reliability (*r* = 0.92-0.97) and internal consistency (α = 0.72-0.89).^39^ The score (of 100) was recorded.

#### Numeric Pain Rating Scale

The levels of neck pain and headache were captured separately with an NPRS. The NPRS has a moderately reliable ICC of 0.76^32^ and a clinically important difference of 13%.^7^ The score is recorded on a Likert scale ranging from 0 (no pain) to 10 (the worst pain ever felt).

### Statistical Analysis

The SRM and ES were determined for the PCSS, NDI, DHI, HDI, and NPRS questionnaires. Only participants who improved (≥1 on the GRC) were considered because SRM statistics implied that all included participants needed to change in the same direction.^22^ For questionnaires other than the PCSS, participants who rated 0 as the baseline score on the PCSS for the symptom assessed by the questionnaire were removed from the analysis (ie, participants who rated 0 for neck pain were removed for analysis of NPRS neck pain and NDI, those who rated 0 for headache were removed for analysis of NPRS headache and HDI, and those who rated 0 for dizziness were removed for analysis of DHI). The ES and SRM were considered large if they were >0.8, moderate if they were between 0.5 and 0.8, and small if they were <0.5.^8^

For MCID calculation, patients with ≤4 on the GRC question were considered “stable or minimally improved,” while patients with ≥5 were considered “greatly improved.” We used the independent *t* test for continuous variables and the chi-square test for nominal variables to compare baseline data and characteristics between the “greatly improved” and “stable or minimally improved” groups; between-group comparisons also included change scores (baseline score minus final score) on the PCSS.

The ROC curve method was used to calculate the MCID. The ROC curve was drawn to determine which PCSS score best differentiated between the “greatly improved” and “stable or minimally improved” groups. The sensitivity (true-positive) values were plotted on the *y*-axis against the 1 – specificity (false-positive) values on the *x*-axis to distinguish between the 2 subgroups of patients. The AUC and 95% CI were used to quantitatively evaluate the ability of the PCSS to correctly distinguish between the 2 subgroups of patients.^12^ The discriminative ability of the questionnaire was deemed excellent for AUC between 0.9 and 1.0; very good between 0.8 and 0.9; good between 0.7 and 0.8; sufficient between 0.6 and 0.7; and poor below 0.6.^38^ The MCID was determined by the optimal cutoff value that corresponded with the maximized average of sensitivity and specificity represented by the uppermost left-hand corner of the ROC curve.^17^

Longitudinal validity was calculated with a Pearson correlation coefficient between change scores on all questionnaires. Participants who rated 0 as the baseline score on the PCSS for a specific symptom were removed from the longitudinal validity analysis of the same symptom-related questionnaire.

## Results

A total of 109 participants were recruited, and no participants were lost to follow-up (see Table 1 for baseline characteristics). For ES and SRM calculations, 14 participants were stable (0 on GRC) at week 6 and removed from the analysis, leaving 95 participants for the calculation of the PCSS SRM and ES. Of the 95 improved participants, 10 did not perceive dizziness at baseline, leaving 85 participants eligible for the DHI; 4 did not perceive headache at baseline leaving, leaving 91 participants for the NPRS headache and HDI; and 10 did not perceive neck pain at baseline, leaving 85 participants eligible for NPRS neck pain and NDI. For the MCID, 37 participants were classified as “stable or minimally improved” (≤4 on GRC) and 72 were classified as “greatly improved” (≥5 on GRC) at week 6. There was no difference between the “greatly improved” and “stable or minimally improved” groups for baseline characteristics and initial scores on PCSS questionnaires (*P* > .05).

**Table 1:** Baseline Scores on Questionnaires*^a^*

	Total (N = 109)	Stable or Minimally Improved (n = 37)	Greatly Improved (n = 72)	*P*
Age, y	39.21 ± 14.00	40.25 ± 14.81	38.69 ± 13.66	.589
Female sex, %	72.7	67.6	76.4	.324
Sports-related injury, %	44.9	40.0	47.1	.488
Days since injury*^b^*	45.06 ± 28.40	53.50 ± 26.57	40.65 ± 28.51	.027*^c^*
No. of previous concussions	1.48 ± 2.29	1.91 ± 2.60	1.27 ± 2.12	.183
PCSS–Total	56.65 ± 23.67	58.78 ± 22.72	55.56 ± 24.22	.503
PCSS-NS	17.72 ± 3.74	18.11 ± 3.74	17.51 ± 3.75	.435
DHI	41.65 ± 21.99	46.92 ± 21.45	38.94 ± 21.92	.073
HDI	41.67 ± 22.14	42.49 ± 22.33	41.25 ± 22.18	.784
NDI	35.42 ± 14.10	39.00 ± 13.74	33.58 ± 14.01	.057
NPRS-NP	3.01 ± 1.96	3.27 ± 1.98	2.88 ± 1.95	.321
NPRS-H	3.67 ± 1.93	4.03 ± 1.85	3.49 ± 1.95	.166

*^a^* Data are presented as mean ± SD unless otherwise indicated. Boldface *P* value indicates a statistically significant difference between stable/minimally improved and greatly improved (*P* < .05). DHI, Dizziness Handicap Inventory; GRC, global rating of change; HDI, Headache Disability Inventory; NDI, Neck Disability Index; NPRS-H, Numeric Pain Rating Scale for headache; NPRS-NP, Numeric Pain Rating Scale for neck pain; PCSS-NS, number of symptoms on Post-Concussion Symptom Scale; PCSS–Total, total score on the Post-Concussion Symptom Scale.

*^b^* Number of days between study enrollment and injury.

*^c^* Statistically significant difference between study groups (*P* < .05).

### Internal Responsiveness

The PCSS number of symptoms (ES, 1.84; SRM, 1.37), PCSS total score (ES, 1.48; SRM, 1.72), DHI (ES, 0.92; SRM, 1.02), HDI (ES, 0.86; SRM, 0.93), NDI (ES, 1.06; SRM, 1.06), NPRS headache (ES, 1.20; SRM, 1.20), and NPRS neck pain (ES, 1.02; SRM, 1.06) were all highly responsive (Table 2).

**Table 2:** Responsiveness of Questionnaires After a Rehabilitation Program*^a^*

	No. of Participants	ES	SRM (95% CI)
PCSS–Total	95	1.48	1.72 (1.40-2.03)
PCSS-NS	95	1.84	1.37 (1.09-1.66)
DHI	85	0.92	1.02 (0.75-1.28)
HDI	91	0.86	0.93 (0.69-1.18)
NDI	85	1.06	1.06 (0.80-1.33)
NPRS-NP	85	1.02	1.06 (0.79-1.33)
NPRS-H	91	1.20	1.20 (0.91-1.45)

*^a^* DHI, Dizziness Handicap Inventory; ES, effect size; HDI, Headache Disability Inventory; NDI, Neck Disability Index; NPRS-H, Numeric Pain Rating Scale of headache; NPRS-NP, Numeric Pain Rating Scale of neck pain; PCSS-NS, number of symptoms on Post-Concussion Symptom Scale; PCSS–Total, total score on the Post-Concussion Symptom Scale; SRM, standardized response mean.

### External Responsiveness

**Figure 1.**
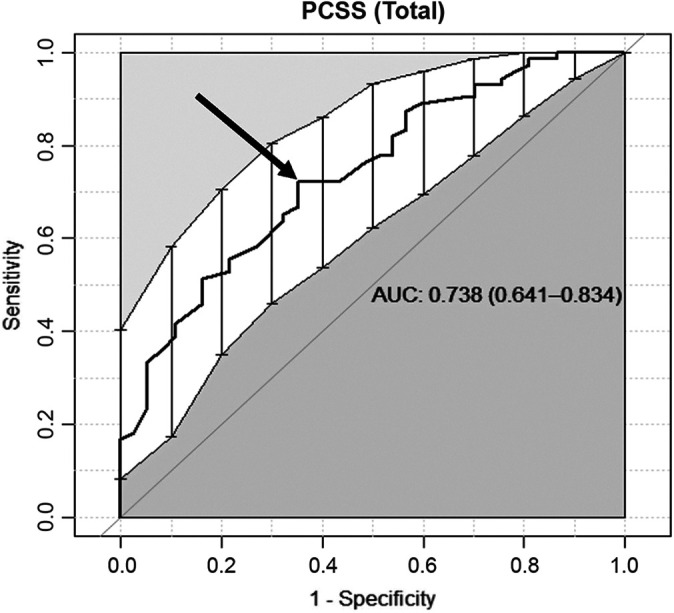
The receiver operating characteristic (ROC) curve of the Post-Concussion Symptom Scale (PCSS) total score. The sensitivity (0.72) and 1 – specificity (0.65) of the minimal clinically important difference curve (arrow) demonstrated an area under the ROC curve (AUC) of 0.738 (95% CI, 0.641-0.834).

**Figure 2.**
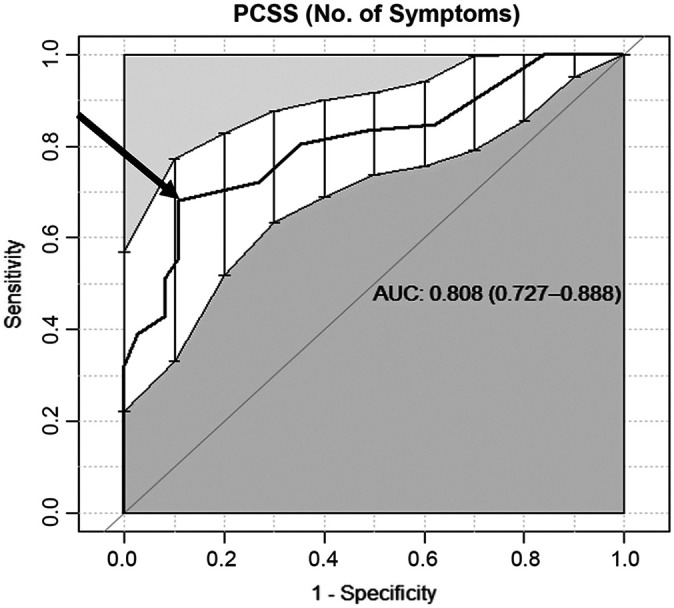
The receiver operating characteristic (ROC) curve of the Post-Concussion Symptoms (PCSS) number of symptoms. The sensitivity (0.68) and 1 – specificity (0.89) of the minimal clinically important difference curve (arrow) demonstrated an area under the ROC curve (AUC) of 0.808 (95% CI, 0.727-0.888).

The AUC was good for the PCSS total score (0.74) and very good for the PCSS number of symptoms (0.81) (Figures 1 and 2). The MCID of the PCSS total score was 26.5 of 132 (95% CI, 14.5-45.5; sensitivity, 0.72; specificity, 0.65) and 5.5 of 22 (95% CI, 3.5-7.5; sensitivity, 0.68; specificity, 0.89) for the number of symptoms. Finally, the change scores on the PCSS total score and number of symptoms were significantly different (*P* < .001) between the “stable or minimally improved” group and the “greatly improved” group in favor of the “greatly improved” group. The mean differences between groups were 18.7 (95% CI, 10.5-26.9) for total score and 5.5 (95% CI, 3.7-7.3) for number of symptoms (Table 3).

**Table 3:** MCID of the PCSS*^a^*

	No. of Participants	MCID (95% CI)	AUC	Sensitivity	Specificity
PCSS–Total	109	26.5 (14.5-45.5)	0.738	0.72	0.65
PCSS-NS	109	5.5 (3.5-7.5)	0.808	0.68	0.89

*^a^* AUC, area under the receiver operating characteristic curve; MCID, minimal clinically important difference; PCSS-NS, number of symptoms on Post-Concussion Symptom Scale; PCSS–Total, total score on the Post-Concussion Symptom Scale.

### Longitudinal Validity

Significant small to moderate correlations were observed between change scores on the PCSS total score and the change score of all other questionnaires (Table 4). Significant small to moderate correlations were found between the PCSS number of symptoms and the DHI, HDI, and NDI. The PCSS number of symptoms was not correlated with the NPRS headache and neck pain.

**Table 4:** Correlation of Change Scores Between Different Questionnaires*^a^*

	PCSS–Total	PCSS-NS
PCSS-NS	0.526*^b^*	—
DHI	0.512*^b^*	0.397*^b^*
HDI	0.437*^b^*	0.474*^b^*
NDI	0.495*^b^*	0.506*^b^*
NPRS–NP	0.235*^c^*	0.169
NPRS–H	0.318*^b^*	0.183

*^a^* DHI, Dizziness Handicap Inventory; HDI, Headache Disability Inventory; NDI, Neck Disability Index; NPRS-H, Numeric Pain Rating Scale of headache; NPRS-NP, Numeric Pain Rating Scale of neck pain; PCSS–NS, number of symptoms on Post-Concussion Symptom Scale; PCSS–Total, total score on the Post-Concussion Symptom Scale. Dash indicates not applicable.

*^b^* Statistically significant difference (*P* < .001).

*^c^* Statistically significant difference (*P* < .05).

## Discussion

This study demonstrated that the PCSS is highly responsive to assess change in patients with persistent symptoms after a concussion (ES and SRM, >1.3), confirming our first hypothesis. Clinicians can use a score of 26.5/132 points (20%) as the MCID of the PCSS total score and of 5.5/22 (25%) as the MCID of the PCSS number of symptoms. The AUCs were >0.7 for the PCSS total score and >0.8 for the PCSS number of symptoms, which confirmed our second hypothesis. All correlations between change scores of questionnaires were positive, and low to moderate, except for the NPRS, which was not correlated with the PCSS number of symptoms. Correlations were around 0.5 between PCSS and NDI, HDI, and DHI. Our third hypothesis was refuted even if correlations tended toward 0.5. This could be explained by the nature of the 3 questionnaires that are mostly related to 1 specific symptom in contrast to the PCSS evaluating 22 different symptoms. The noncorrelation between change scores of NPRS and the number of symptoms on PCSS was expected because NPRS exclusively assesses the intensity of 1 symptom so it has 1 dimension, whereas PCSS evaluates multiple symptoms.^19^ Our hypothesis for NPRS correlations was thus confirmed. Our exploratory responsiveness analysis demonstrated that when headache, neck pain, and/or dizziness are present, NPRS and symptom-related questionnaires (DHI, HDI, and/or NDI) can be considered highly responsive in this population.

### External Responsiveness/MCID

Evidence is limited concerning the psychometric properties of symptom checklists for concussion/mild traumatic brain injury.^1^ To our knowledge, this is the first study to demonstrate the responsiveness of the PCSS. An MDC of 12.3 points had already been established for the PCSS total score, while the MDC for the number of symptoms has not yet been established. The MDC represents the raw score of the measure that needs to be reached to obtain a change that is superior to the measurement error.^34^ The MCID of the PCSS total score calculated with our cohort exceeds the MDC, so it can be used with confidence by the clinician as the clinical meaningful change score over time. Clinically, it can also be used to set intervention goals that are relevant to the patient.

The MCID reflects the status that is associated with patient-perceived meaningful improvement.^12^ An MCID of 5.5 points on the number of symptoms rated in the PCSS means that if a patient improves ≥6 on the scale, the change is a clinically relevant improvement. For example, our finding suggests that if a patient rated 15 symptoms at the initial evaluation and 9 symptoms at the 6-week follow-up assessment after an intervention program, the clinician could state that the 6-symptom improvement is meaningful for the patient. However, the specificity and sensitivity of the PCSS total score and number of symptoms MCID were 0.65 and 0.72, and 0.89 and 0.68, respectively. Except for the specificity of the PCSS number of symptoms, other values were lower than expected. Specificity refers to the capacity of the measure to correctly identified a true negative. Concretely, it means that when an improvement was defined as <5.5 symptoms on the PCSS, 89% of the patients were correctly identified as “stable or minimally improved.” Sensitivity refers to the capacity of a test to detect a true positive, which means that when patients rated >5.5 symptoms on the PCSS, 68% of the patients were correctly identified as greatly improved. So, if a patient rates >6 points on the PCSS number of symptoms, there is 32% chance that the patient would still rate his or her condition as minimally improved.

### Longitudinal Validity

The longitudinal validity refers to the extent to which changes on one measure correlate with changes on another measure. According to the COSMIN guideline,^35^ a correlation <0.3 means that instruments measure an unrelated construct, which was the case for the correlation between change on the NPRS and change on the PCSS. The NPRS on neck pain could be a measure of a neck-related injury concomitant to the concussion (ie, whiplash injury), which could be a supplemental explanation for the weak correlation. A correlation between 0.3 and 0.5 implies that the constructs are dissimilar but related. Since correlations between PCSS and NDI, HDI, and DHI are low to moderate, clinicians could supplement the PCSS with disability questionnaires when they want to assess the evolution of the condition in time in patients with headache, neck pain, or dizziness.

### Limitations

In this study, the only concussion-specific checklist questionnaire studied was the PCSS. However, other checklist questionnaires have been developed for this population. These questionnaires evaluate between 10 and 25 symptoms and use a 5- to 7-point Likert scale to score each symptom.^28^ Some questionnaires like the Rivermead Post-Concussion Symptoms Questionnaire and the 21-Item ImPACT (Immediate Post-Concussion Assessment and Cognitive Testing) have been studied for their reliability and validity^2,28,33^ but not for their responsiveness. Future studies should therefore look at the responsiveness of these questionnaires.

The use of the GRC as the external criterion could introduce a recall bias, as participants may not have remembered at week 6 how they were at baseline. Also, GRC has never been validated in the population after a concussion, which is recommended for an external criterion when assessing responsiveness.^35^ Regarding our decision to include participants with a GRC rating of 4 (ie, moderately improved) in the “stable or marginally improved” group, one could state that it could fall in either the “greatly improved” or the “stable or minimally improved” group. However, our results show a successful delineation between both groups confirmed by the significant between-group difference of change scores in favor of the improved group. This difference increases our confidence that the determined cutoff value of GRC is an adequate external criterion.^11,42^

The use of questionnaires mainly evaluating the physical dimension of concussion is a limitation that narrows the interpretation of the longitudinal validity analysis to the physical aspect of concussion. The PCSS also assessed other dimensions of concussion (affective, cognitive, sleep arousal). Specific questionnaires on those dimensions could have been included in the present study and would have enriched the longitudinal validity analysis.

The limited number of participants in the “stable or minimally improved” group (n = 37) could be seen as a weakness. The COSMIN guideline suggests a minimum sample size of 100 participants (50 per group).^31^ Our total sample size exceeds the recommended 100 participants. However, it is possible that the significantly smaller “stable or minimally improved” group contributed to the low sensitivity and specificity of the MCIDs. The main reason why the greatly improved group was larger in comparison with the “stable or minimally improved” group is that natural evolution of concussion is generally favorable after a rehabilitation program containing aerobic exercises and education.^21^

## Conclusion

This study fills a significant knowledge gap by reporting the responsiveness of diverse questionnaires frequently used when symptoms persist after a concussion. Our findings demonstrate that the PCSS is highly responsive to change, with an MCID of 26.5 points for the total score and of 5.5 symptoms for the number of symptoms. Other questionnaires frequently used to assess the most reported physical symptoms after a concussion were also shown to be highly responsive. As this is the first study on the responsiveness of such questionnaires, further studies on different cohorts (eg, acute or chronic) are necessary to confirm our results and improve knowledge on psychometric properties of questionnaires used in clinical practice after a concussion.
